# A new method to customize protein expression vectors for fast, efficient and background free parallel cloning

**DOI:** 10.1186/1472-6750-13-12

**Published:** 2013-02-14

**Authors:** Judith Scholz, Hüseyin Besir, Claudia Strasser, Sabine Suppmann

**Affiliations:** 1 Max-Planck Institute of Biochemistry, Am Klopferspitz 18, 82152, Martinsried, Germany; 2 EMBL Heidelberg, Meyerhofstrasse 1, 69117, Heidelberg, Germany

**Keywords:** Parallel cloning, Restriction enzyme free cloning, Expression screening, Bacterial expression, Insect cell expression, Mammalian expression, Protein production

## Abstract

**Background:**

Expression and purification of correctly folded proteins typically require screening of different parameters such as protein variants, solubility enhancing tags or expression hosts. Parallel vector series that cover all variations are available, but not without compromise. We have established a fast, efficient and absolutely background free cloning approach that can be applied to any selected vector.

**Results:**

Here we describe a method to tailor selected expression vectors for parallel **S**equence and **L**igation **I**ndependent **C**loning. SLIC cloning enables precise and sequence independent engineering and is based on joining vector and insert with 15–25 bp homologies on both DNA ends by homologous recombination. We modified expression vectors based on pET, pFastBac and pTT backbones for parallel PCR-based cloning and screening in *E.coli*, insect cells and HEK293E cells, respectively. We introduced the toxic *ccdB* gene under control of a strong constitutive promoter for counterselection of insert less vector. In contrast to DpnI treatment commonly used to reduce vector background, *ccdB* used in our vector series is 100% efficient in killing parental vector carrying cells and reduces vector background to zero. In addition, the 3’ end of *ccdB* functions as a primer binding site common to all vectors. The second shared primer binding site is provided by a HRV 3C protease cleavage site located downstream of purification and solubility enhancing tags for tag removal. We have so far generated more than 30 different parallel expression vectors, and successfully cloned and expressed more than 250 genes with this vector series. There is no size restriction for gene insertion, clone efficiency is > 95% with clone numbers up to 200. The procedure is simple, fast, efficient and cost-effective. All expression vectors showed efficient expression of eGFP and different target proteins requested to be produced and purified at our Core Facility services.

**Conclusion:**

This new expression vector series allows efficient and cost-effective parallel cloning and thus screening of different protein constructs, tags and expression hosts.

## Background

**Figure 1:**
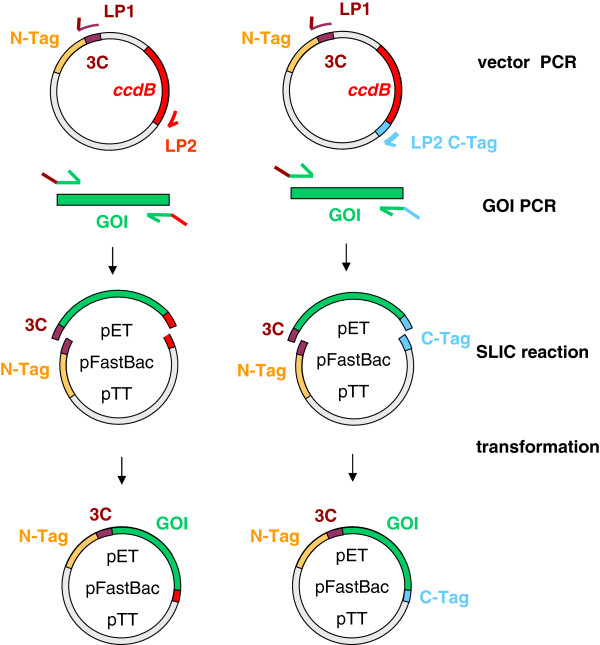
**Principle of parallel SLIC cloning with negative *ccdB* selection.** The vector is PCR linearized with LP1 forward and LP2 reverse primer. The LP1 primer corresponds to PreScission protease site (3C) for tag removal. The LP2 primer is either located at the C-terminus of *ccdB* or corresponds to a C-terminal tag. In both cases the *ccdB* gene is deleted upon PCR amplification thereby allowing counterselection of parental empty vector in *ccdB* sensitive cells. The **G**ene **o**f **I**nterest (GOI) is PCR amplified with primers composed of 5’ and 3’ gene specific sequences plus 15 bp – 25 bp extensions complementary to LP1 and LP2 vector primers, respectively.

As central Core Facility Labs at the Max-Planck Institute of Biochemistry and the EMBL we provide in-house services for recombinant protein production. The proteins we are asked to produce are from various sources and protein families and are used for crystallization, immunization, biochemical, biophysical or biological studies. We perfom in-depth protein analysis to ensure that delivered proteins are properly folded. However, in many cases, this is a fairly challenging task to achieve, despite the many options that can improve proper protein folding such as construct design, solubility enhancing fusion tags, expression conditions, expression hosts and improved protein purification protocols. The constant challenge is to identify a successful combination of parameters with a minimum of resources. Apart from unbiased HTP approaches [1] or targeted selection [2,3], it still remains a time consuming trial and error process in most non-automated protein labs. We had initially focused our screening efforts on constructs and solubility tags in *E. coli* as a first choice. Eukaryotic hosts were used if suggested by the literature or previous experience, or in case *E. coli* screening had failed. As this happened frequently we decided to implement parallel testing of constructs, solubility tags and expression hosts altogether. In order to handle all these different expression constructs, an efficient parallel cloning method was required. In the past few years, a number of powerful combinatorial cloning methods have been introduced. Gateway technology (Life Technologies) opened the area of combinatorial cloning more than a decade ago. More recent additions to the list are type II restriction enzymes (Stargate, IBA, Germany), Golden Gate Shuffling [4], RF cloning [5] and **S**equence and **L**igation **I**ndependent **C**loning SLIC [6]. For recombinant protein expression, several commercial (Novagen, IBA, Life Technologies) and non-commercial [7] parallel vector series are available. However, we had already established different expression systems with their respective vectors: The *E.coli* pETM vector series have different tags followed by a protease cleavage site and are based on the same pET backbone [8]. Differences in expression levels are exclusively based on different tags, not on backbones differences such as copy number, spacer sequences or others. Expression uses the powerful T7 promoter system at low vector copy number and plasmids confer Kanamycin resistence instead of Ampicillin, which is crucial for plasmid stability [9], particularly in high cell density fermentations. Transient transfection of mammalian cells is another efficient and fast method for protein production. The HEK293 (human embryonic kidney) cell line is widely used due to high transfection efficiency and suspension growth in serum-free media. A variety of HEK293 cells are currently available with significant differences in productivity [10]. The Epstein-Barr virus nuclear antigen 1 (EBNA1) in HEK293E cell line interacts with oriP on pTT vectors which increases plasmid persistence and protein expression levels [11]. Therefore we preferred to customize pETM, pFastBac and pTT vectors for parallel cloning rather than adapt to a new system. The method must be sequence and restriction-site independent to allow for incorporation of any DNA fragment into any of the vectors. It must be directional and precise and most important, it must be fast, simple, efficient and cost-effective. SLIC cloning enables sequence independent, precise cloning with minimal or no changes in the amino acid sequence of the target protein and is based on homologous recombination of vector and insert with 15–25 bp homologies on both DNA ends. The reaction is enhanced using either T4 DNA polymerase, recA protein or incomplete PCR products [6]. In order to adapt vectors for SLIC, they need to share a common stretch of nucleotides at both ends of the linearized plasmid. We have chosen the HRV 3C protease [12] cleavage site and the 3’ end of the toxic *ccdB* gene to serve as primer binding sites common to all of the new vectors (Figure 1). The HRV 3C recognition site is located downstream of the N-terminal purification or solubility enhancing tag and can be used for tag removal. The CcdB protein inhibits bacterial growth by selective inhibition of *E. coli* DNA gyrase and can be neutralized by the antitoxin *ccdA*. The ccdB technology was developed by Delphi Genetics [13] and is licenced for use in Gateway vectors as well (Life Technologies). In the SLIC strategy presented here, *ccdB* introduced into the vector series was designed for strong constitutive expression in order to suppress growth in non-resistant cells at 100% efficiency. The vector is used as a PCR template for the amplification of the linear vector fragment, where *ccdB* is deleted. The *ccdB* gene on the template thus prevents the carry-over of the original vector during purification and SLIC reaction by preventing growth of colonies not containing the gene of interest. We developed pET, pFastBac and pTT parallel cloning vectors, named pCoofy_1-x_ and present protein expression data in each of the respective host organisms.

## Results and discussion

### Vector design and cloning strategy

**Figure 2:**
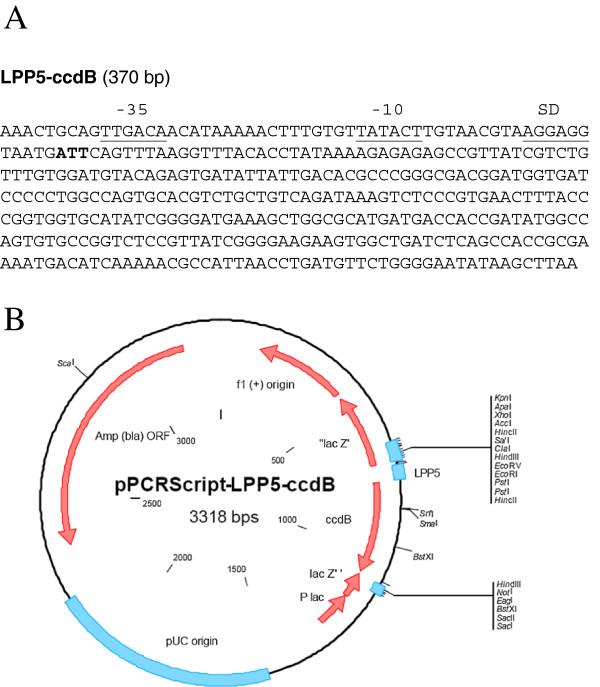
***ccdB* cassette used for pCoofy vectors.** (**A**) The *Llp5* variant of the strong *E.coli* major outer membrane lipoprotein promoter was introduced upstream of the coding sequence to drive constitutive *ccdB* transcription. -35 and -10 regulatory sequences are highlighted. A Shine-Dalgarno sequence was also added to ensure *ccdB* translation. The original start codon was converted to ATT (Ile, indicated in bold) for technical reasons. (**B**) The *ccdB* cassette was delivered by Sloning in pPCRSript (sequence available on request) and tested for toxicity by transformation into sensitive *E.coli* cells.

**Figure 3:**
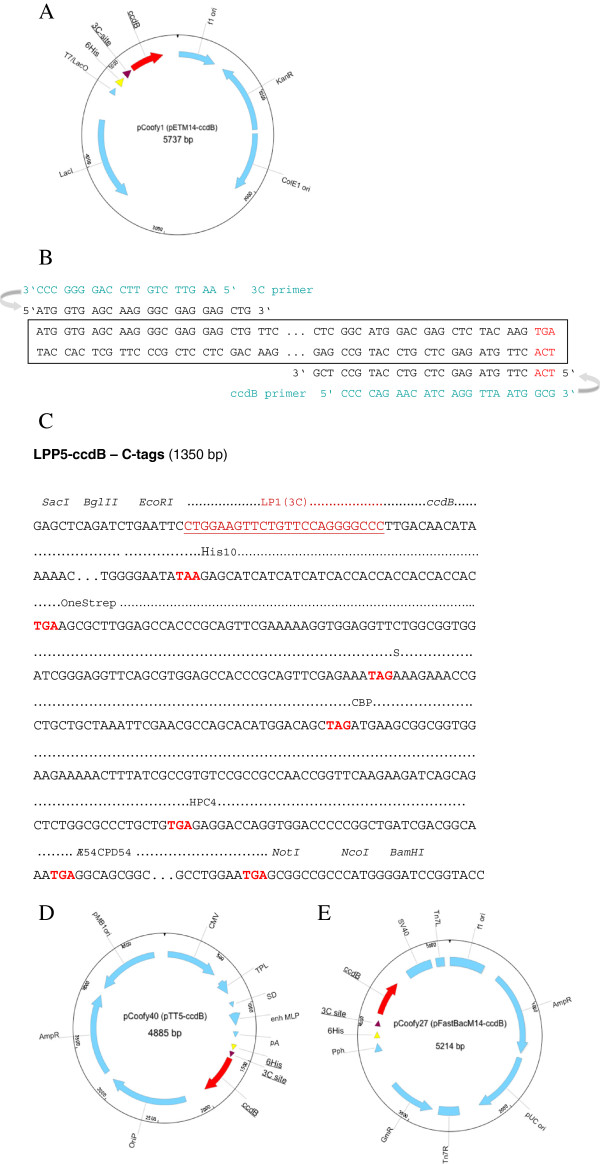
**Representative maps of parallel *ccdB* vectors for protein expression in *E. coli*, Baculovirus and HEK293E.** For each of these hosts, one example of the pET (*E. coli*) (**A**), pFastBac (Baculovirus) (**E**) and pTT (HEK293E) (**D**) ccdB vector series is shown. All three backbones share common LP1 (3C) and LP2 (*ccdB*) primer binding sites for parallel cloning. (**B**) Primer design is illustrated for the universal PreScission 3C-ccdB primer pair of the pCoofy vector series. Gene specific primer sequences are fused to sequence overhangs of 20–24 nucleotides which are complementary to the corresponding vector amplification primer (see Table 2). (**C**) **2**^**nd**^**generation*ccdB*cassette including C-tags** The *ccdB* cassette as shown in Figure 2A starting at TTGACA (−35 region) was fused to a row of C-terminal tags each separated by a stop codon. LP1 (3C) sequence was added upstream of the *ccdB* coding sequence. Restriction sites at both ends were also added for subsequent cloning into different vector backbones. The complete cassette was synthesized by GeneArt (now part of Life Technologies) with concomitant codon optimization of the tags for eukaryotic expression.

In order to drive strong constitutive *ccdB* expression from pCoofy vectors, we used the promoter of the major outer membrane lipoprotein *OmpA*, which is one of the strongest promoters in *E. coli*[14]. We inserted the respective *llp5* promoter variant and a Shine-Dalgarno sequence upstream of the *ccdB* coding sequence (Figure 2A) to ensure translation. pPCRScript-LPP5-ccdB (Figure 2B) and all pCoofy *ccdB* derivatives show 100% killing efficiency when transformed into non-resistant cells. Occasionally, we observed the occurrence of *ccdB* inactivation during plasmid propagation in *ccdB* Survival™ cells (Life Technologies) under high selective pressure such as plasmids with high copy number. Therefore killing activity has to be verified for every single batch of vector DNA. However, since we use the vectors only as templates for PCR linearization the amount of DNA used for a single PCR is very low and usually sufficient for multiple SLIC reactions. Typically, 1 ng plasmid DNA is needed for a single SLIC reaction. We first cloned Llp5-ccdB into pETM14, pETM22, pETM33 and pETM44 to generate pCoofy1, 2, 3 and 4 (Figure 3A and Table 1). The parallel SLIC cloning procedure was established using eGFP as gene of interest according to the strategy illustrated in Figure 1A. pCoofy vectors were PCR linearized with 3C - LP1 forward and ccdB - LP2 reverse primer (Table 2). The LP1 primer corresponds to the HRV 3C protease site , the LP2 primer is located at the C-terminus of *ccdB* in order to delete the gene upon PCR amplification. eGFP was PCR amplified with primers composed of gene specific sequences plus 20 bp and 25 bp extensions complementary to LP1 and LP2 vector primers, respectively (Figure 3B).

**Table 1:** Current list of pCoofys

**pCoofy**	**parental vector**	**N-tag**	**C-tag**	**Host**	**fd primer**	**rev primer**
pCoofy1	pETM14	His6	none	*E. coli*	3C	ccdB
pCoofy18	pCoofy1	His10	none	*E. coli*	3C	ccdB
pCoofy7	pETM14	S	none	*E. coli*	3C	ccdB
pCoofy12	pCoofy1	OneStrep	none	*E. coli*	3C	ccdB
pCoofy34	pCoofy12	S-OneStrep	none	*E. coli*	3C	ccdB
pCoofy21	pCoofy12	His10-OneStrep	none	*E. coli*	3C	ccdB
pCoofy19	pCoofy1	CBP	none	*E. coli*	3C	ccdB
pCoofy2	pETM22	Trx-His6	none	*E. coli*	3C	ccdB
pCoofy38	pCoofy2	Trx-His10	none	*E. coli*	3C	ccdB
pCoofy3	pETM33	His6-GST	none	*E. coli*	3C	ccdB
pCoofy8	pETM14	Halo	none	*E. coli*	3C	ccdB
pCoofy35	pCoofy4	MBP	none	*E. coli*	3C	ccdB
pCoofy4	pETM44	His6-MBP	none	*E. coli*	3C	ccdB
pCoofy15	pETM14	NusA	none	*E. coli*	3C	ccdB
pCoofy16	pCoofy15	His10-NusA	none	*E. coli*	3C	ccdB
pCoofy5	pET28M-Sumo1	His6-Sumo1	none	*E. coli*	Sumo1	ccdB
pCoofy6	pET28M-Sumo3	His6-Sumo3	none	*E. coli*	Sumo3	ccdB
pCoofy17	pCoofy6	His10-Sumo3	none	*E. coli*	Sumo3	ccdB
pCoofy22	pCoofy7	S	His10	*E. coli*	3C	10His
pCoofy31	pCoofy12	OneStrep	His10	*E. coli*	3C	10His
pCoofy36	pCoofy34	S-OneStrep	His10	*E. coli*	3C	10His
pCoofy24	pCoofy19	CBP	His10	*E. coli*	3C	10His
pCoofy23	pCoofy14	Trx	His10	*E. coli*	3C	10His
pCoofy25	pCoofy8	Halo	His10	*E. coli*	3C	10His
pCoofy37	pCoofy35	MBP	His10	*E. coli*	3C	10His
pCoofy26	pCoofy15	NusA	His10	*E. coli*	3C	10His
pCoofy32	pCoofy1	His6	OneStrep	*E. coli*	3C	OneStrep
pCoofy33	pCoofy18	His10	OneStrep	*E. coli*	3C	OneStrep
pCoofy11	pIEX1	His10	none	Insect	3C	ccdB
pCoofy27	pFastBac	His6	none	Insect	3C	ccdB
pCoofy28	pFastBac	His6-GST	none	Insect	3C	ccdB
pCoofy29	pFastBac	His6-MBP	none	Insect	3C	ccdB
pCoofy40	pTT5	none	flexible	HEK293E	3C	flexible

**Table 2:** Primer for vector amplification and complementary primer extensions for gene of interest

**LP1 forward vector primer**	
3C	5' GGGCCCCTGGAACAGAACTTCCAG 3'
Sumo1	5' TCCACCGGTTTGTTCCTGGTAGAC 3'
Sumo3	5' TCCACCGGTCTGCTGCTGGAACAC 3'
N-tagless pET	5' GGTATATCTCCTTCTCTAGAGGGGAATTGTTATCCGCTC 3'
N-tagless pFastBac	5' GGAATTCCGCGCGCTTCGGACC 3'
**LP2 reverse vector primer**	
ccdB	5' CGCCATTAACCTGATGTTCTGGGG 3'
10His	5' GAGCATCATCATCATCACCAC 3'
OneStrep	5' AGCGCTTGGAGCCACCCGCAG 3'
S	5' AAAGAAACCGCTGCTGCTAAATTCG 3'
HPC4	5' GAGGACCAGGTGGACCCCCGG 3'
**LP1 forward gene primer**	
3C	5' AAGTTCTGTTCCAGGGGCCC – GOI seq 3'
Sumo1	5' GTCTACCAGGAACAAACCGGTGGA – GOI seq 3'
Sumo3	5' GTGTTCCAGCAGCAGACCGGTGGA – GOI seq 3'
**LP2 reverse gene primer**	
ccdB	5' CCCCAGAACATCAGGTTAATGGCG - Stop - GOI - seq 3'
10His	5' GTGGTGATGATGATGATGCTC - Stop - GOI - seq 3'
OneStrep	5' CTGCGGGTGGCTCCAAGCGCT - Stop - GOI - seq 3'
S	5' AGCAGCAGCGGTTTCTTT - Stop - GOI - seq 3'
CBP	5' CTTCCACCGCCGCTTCATC - Stop - GOI - seq 3'
HPC4	5' GGGGTCCACCTGGTCCTC – Stop - GOI - seq 3'
Æ54CPD54	5' CAGGATCTTGCCGCTGCC – Stop - GOI - seq 3'

When the SLIC reaction was carried out with insert and vector at a molar ratio of 1:3 without any treatment prior to transformation into chemocompetent OmniMAX™ 2 T1^R^ cells, cloning efficiency was below 70%. Addition of recA raised overall cloning efficiency to > 95%. T4 DNA Polymerase treatment of vector and insert [6] was equally efficient, but due to simplicity we continued with the recA protocol. We tested several other variations to the basic protocol, however none of these further improved cloning efficiency: number of PCR cycles, PCR without extension step, extended LP1 and LP2 primer length for vector and insert PCR amplification, amount of vector and insert, molar ratio of vector and insert, 5 min 95°C denaturation of vector and insert mix followed by slow renaturation at 22° (data not shown). *E.coli* cells used for transformation may have an impact on quantity and quality of recombination events and should be tested first. At the Max-Planck Institute we use chemocompetent OmniMAX™ 2 T1^R^ cells with a typical transformation efficiency of 10^7^/μg pUC plasmid DNA.

### Vector list and cloning statistics

The list of *E.coli* pCoofy vectors was extended by modifying additional pETM vectors or by introducing His_10_, OneStrep, S or Halo tags from templates listed in Table 1. All N- terminal tags are followed by the HRV 3C recognition site Leu-Phe-Gln/Gly-Pro. Specific cleavage occurs between Gln and Gly, with Gly-Pro remaining at the N terminus of the target protein. In order to express proteins that have to retain their native N-terminus after tag removal we generated ccdB versions of pET28M-Sumo1 and pET28M-Sumo3 vectors. The SUMO (**S**mall **U**biquitin-like **Mo**difier) tag is recognized and removed by SUMO protease in a structure specific manner to yield the target protein with its native N terminus [15]. Cloning a target gene into pCoofy5 and pCoofy6 requires the corresponding Sumo-LP1 primer for vector and insert PCR amplification (Table 2). We also generated *E.coli* vectors with either a C-terminal His_10_ or OneStrep tag (Table 1), which require the corresponding LP2 vector and insert primer for SLIC cloning (Figure 1, Table 2). Moreover, constructs without any N-terminal tag can be generated using LP1 tagless primers for the appropriate backbone. N-tagless primer were validated and used for pET and pFastBac backbones so far (Table 2). In order to further increase C-tag variations but at the same time reduce the number of vectors to be generated we designed a 2^nd^ generation ccdB cassette (Figure 3C). Llp5-ccdB is followed by a row of C-terminal tags all separated by a stop codon. Depending on the LP2 primer used for vector and insert PCR linearization, either no tag, the His_10_, S, OneStrep, CBP, HPC4 or Æ54CPD54 self cleaving tag is fused to the C-terminus of the protein. Except for the Æ54CPD54 self cleaving tag, C-terminal tags lack a protease cleavage site and cannot be removed. At this time we have cloned this *ccdB* - C- tag cassette into pTT5 (Figure 3D) and validated eGFP and target gene expression in mammalian cells. pCoofy derivatives of pFastBac1 are currently available with His_6_, His_6_GST and His_6_MBP N-terminal tags (Figure 3E, Table 1).

**Figure 4:**
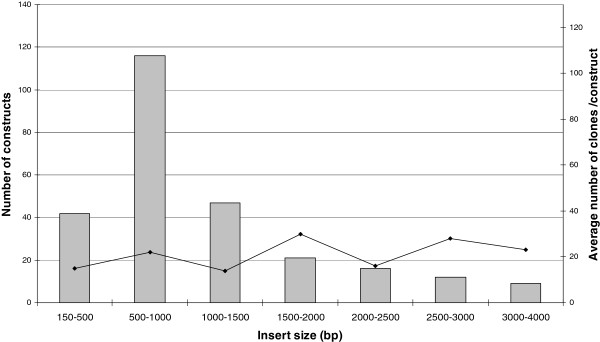
**Cloning statistic.** Typically, three colonies per transformation were selected for plasmid miniprep and sequencing reaction. All of the recombinant constructs listed here were approved to be correct by sequence alignments.

We have effectively cloned more than 250 inserts into all different vectors of the pCoofy series. For all constructs, the DNA sequence of the translated gene fusion was controlled. We did not sequence the vector backbone of recombinant constructs, as we have never observed any compromised vector function. Insert sizes ranged between 150 and 3939 bp, with a majority in the range of 500–1000 bp. The number of clones per SLIC reaction varied with an average of about 20 throughout the entire distribution of insert sizes. As we never observed any background, we were not concerned if clone numbers were low, as the clone was correct in almost all cases (Figure 4).

### Expression in *E.coli*

**Figure 5:**
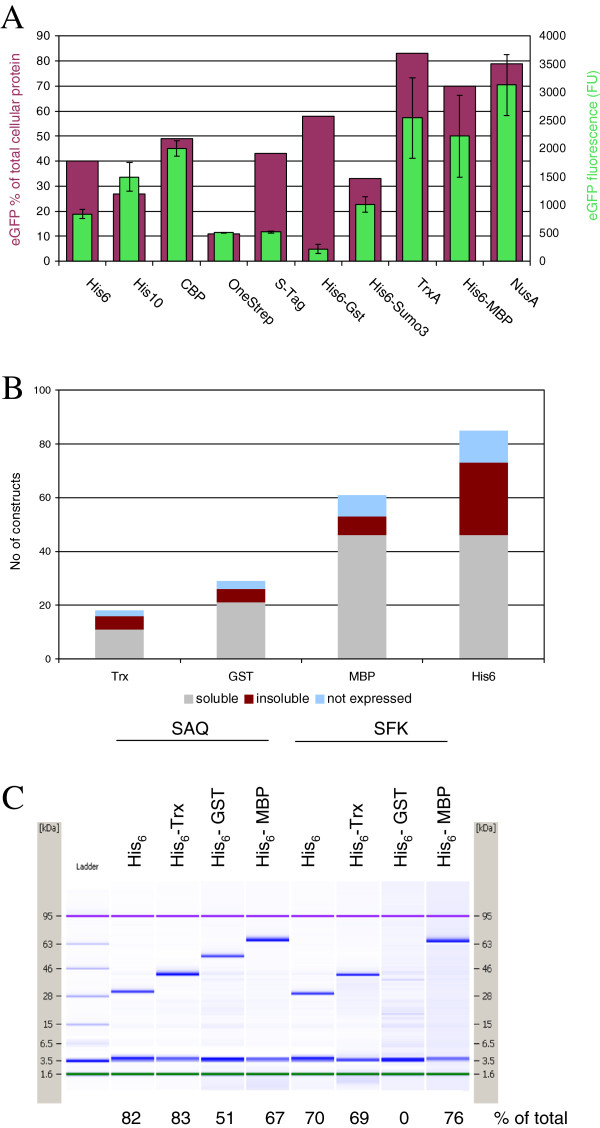
**Expression in *E.coli.*** (**A**) eGFP was SLIC cloned into different *E.coli* pCoofy vectors using standard LP1 (3C) and LP2 (*ccdB*) primers and expressed in BL21 Rosetta (DE3) at 24°C. 2ml cultures were harvested and lyzed in PBS + protease inhibitors at consistent cell density / buffer ratio. eGFP expression level in total lysate was monitored on Agilent BioAnalyzer P80 Chips; eGFP fluorescence in cleared lysate was recorded on a LightCyclerII instrument. (**B**) Expression results for different target proteins cloned in pCoofy1 (His_6_), pCoofy2 (His_6_Trx), pCoofy3 (His_6_GST) and pCoofy4 (His_6_MBP) plus pCoofy35 (MBP only). Proteins are classified as soluble, insoluble or not expressed. “Soluble” proteins could be enriched by IMAC and were further purified with or without tag. Proteins classified as “insoluble” were not soluble in standard IMAC buffers (buffer A). These constructs were either subject to refolding, buffer screens or discontinued. Proteins classified as “not expressed” cover constructs, that were expressed at low or even undetectable level in Western Blot analysis and were thus discontinued. (**C**) Pil1 (UniProt: P53252). SAQ and SFK mutants were SLIC cloned into pCoofy1 (His_6_), pCoofy2 (His_6_Trx), pCoofy3 (His_6_GST) and pCoofy4 (His_6_MBP) using standard LP1 (3C) and LP2 (*ccdB*) primers and expressed in BL21 Rosetta (DE3) at 30°C. oD3 samples (0,1 ml culture of oD_600_ of 3 is lyzed in 50μl sample buffer) were loaded on Agilent BioAnalyzer P80 Chips. Expression levels indicated are based on relative peak quantification.

Prior to use for requested target proteins, every new vector was validated with eGFP for cloning and small scale expression in the respective host. In case the expression level was unexpectedly low, we removed the vector from the list. For example a His_10_Trx-eGFP construct was expressed at about 20% of total protein in *E.coli* total cell lysate. When the double tag was switched to TrxHis_10_-eGFP the expression level increased to more than 70% (data not shown). Figure 5A shows a comparison of expression level and solubility of eGFP fused to several purification and solubility enhancing tags in *E.coli*. Trx, MBP and NusA protein fusions show the highest solubility as reported previously [8] and also the highest expression level at up to 80% of total cellular protein. Most interesting, His_6_GST-eGFP expressed at a high level but at low solubility. This result corroborates our previous observation, that His_6_GST expressed from the original pETM33 vector was insoluble (data not shown). Most of the *E.coli* expression data for requested target proteins at our Protein Production Service were collected for the first pCoofy vectors 1–4 corresponding to N-terminal His_6_, His_6_Trx, His_6_GST and His_6_MBP tags. In agreement with the eGFP expression data, MBP had a major impact on protein solubility (Figure 5B). This is also exemplified by *E.coli* expression of two Pil protein mutants in pCoofy1, 2, 3, and 4 (Figure 5C): expression levels range between 50% - and 80%, of target protein in *E.coli* total cell lysate with lower or no expression of the His_6_GST fusion protein. In the course of the project, only the His_6_MBP-Pil fusion proteins were soluble also after tag removal with HRV 3C protease (data not shown).

### Expression in insect cells

pCoofy vectors allow for parallel screening in *E.coli* and insect and/or mammalian cells which has increased protein production throughput in our facilities substantially. Here we show two examples, Vasp and ODC, where parallel cloning allowed us to switch from the *E.coli* expression host to the Baculovirus expression system easily without much delay. In the case of Topoisomerase 1, Baculovirus expression of two parallel constructs improved project progress.

**Figure 6:**
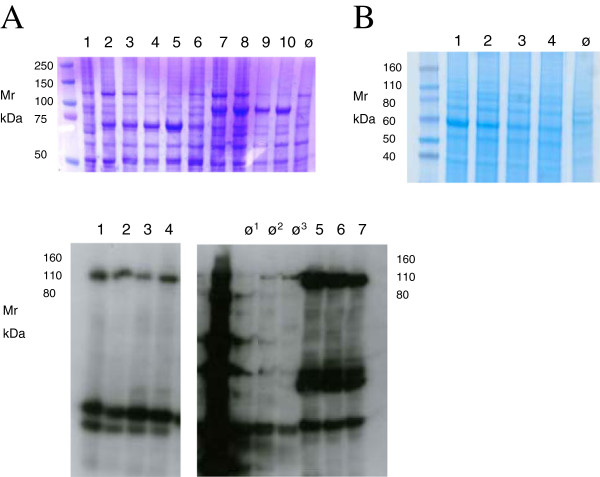
**Baculovirus expression in insect cells.** pFastBac derivatives pCoofy27 (His_6_), pCoofy28 (His_6_GST) or pCoofy29 (His_6_MBP) were transformed into DH10Bac *E.coli* cells to generate recombinant Baculovirus (Bac-to-Bac®, Life Technologies). HighFive or SF9 cells were either infected with P2 virus stock or **B**aculo **I**nfected **I**nsect **C**ells BIIC at dilutions indicated. Cells were harvested after 72h and lyzed in SDS Lämmli buffer. Total lysate was loaded on 4–12% NuPAGE gels (Life Technologies). ø cells infected with non-recombinant Baculovirus. (**A**) Expression of His_6_GST-Vasp (lanes 1 to 5) and His_6_MBP-Vasp (lanes 6 to 10) (UniProt: P50552) in HighFive cells after infection with P2 virus stock; 48 h expression , 2000 fold virus dilution (lanes 1 and 6); 72 h expression at 20.000 (lanes 2 and 7), 2000 (lanes 3 and 8), 200 (lanes 4 and 9) and 40-fold (lanes 5 and 10) virus dilution, respectively; coomassie stained (**B**) Expression of His_6_-ODC (NCBI RefSeq NP_002530) in HighFive cells after infection with BIIC; lanes 1–4 BIIC at 1:500, 1:1000, 1:2000, 1:4000 dilution respectively; coomassie stained (**C**) Expression of Topoisomerase I (UniProt P04786) fused to His_6_GST-topisomerase I (lanes 1 to 4) and His_6_MBP (lanes 5 to 7) in High Five cells. Cells were infected with BIIC at 1:500, 1:1000, 1:2000 (lanes 1 to 3 and 5 to 7), and 1:4000 (lane 4) respectively. ø1 cells infected with insertless virus; ø2 uninfected H5 cells, ø3 uninfected SF9 cells, detected by Western Blot analysis using His-Probe™ reagent (ThermoScientific, Braunschweig, Germany).

Expression of GST-Vasp in *E.coli* was described previously [16]. When we expressed GST-Vasp in *E.coli* we observed partial proteolytical degradation and also coaggregation of degradation products with full-length protein. Instead of investing time in optimizing bacterial expression, we cloned Vasp into pFastBac derivatives pCoofy28 (His_6_GST) and pCoofy29 (His_6_MBP) and expressed both constructs in SF9 cells without any signs of degradation (Figure 6A). Purified full-length His_6_GST-Vasp was shown to be biologically active (data not shown). Ornithin Decarboxylase (ODC) was properly folded when expressed in *E.coli* , but for co-expression purposes it had to be purified from insect cells. We therefore cloned the *ODC* gene into pCoofy27 (His_6_) and expressed the ODC protein in High Five cells (Figure 6B). Again, SLIC cloning enabled rapid change of expression host. Expression of GST-Top1 in insect cells was described previously [17]. We cloned Top1 in pCoofy28 (His_6_GST) and pCoofy29 (His_6_MBP) in parallel and tested expression in High Five cells. His_6_MBP-Top1 showed much higher expression level than His_6_GST-Top1 and was purified to homogeneity in enzymatically active form. (data not shown) (Figure 6C).

### Expression in HEK293E cells

**Figure 7:**
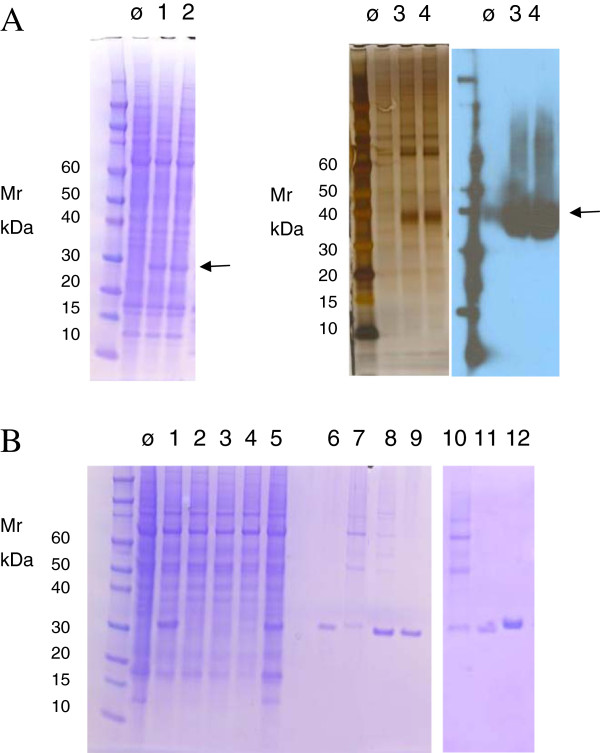
**Expression in HEK293E cells.** (**A**) pTT5-eGFP and pTT22-CD40L (GenBank NT_039706) constructs were generated by restriction enzyme cloning. pCoofy40L-eGFP was generated using standard LP1 (3C) and LP2 (*ccdB*) primers. Coofy40-CD40L was generated using pTT22-CD40L as template for PCR amplification of the insert. Gene specific LP1 primer sequence included vector VEGF signal sequence from pTT22. Recombinant plasmids were transfected into HEK293E cells and expressed for 72h. Total lysates of untransfected cells, pTT5-eGFP (lane 1) pCoofy40-eGFP (lane 2), cell culture supernatants of untransfected cells, pTT5- CD40L (lane 3) and pCoofy40-CD40L (lane 4) detected by Coomassie, silver staining and Western Blot HisProbe™-HRP detection. (**B**) eGFP was SLIC cloned into pTT derivative Coofy40 using standard LP1 (3C) primer in combination with either His_10_, OneStrep, S, CBP, HPC4 LP2 primer to introduce a C-terminal tag. Recombinant plasmids were transfected into HEK293E cells end expressed for 72h. Total lysates of untransfected cells, pCoofy40-eGFP fused to C-terminal S-tag (lane 1), CBP (lane 2), HPC (lane 3), OneStrep (lane 4) and His_10_ (lane 5). Eluates from S-Protein Agarose (lane 6), Calmodulin Affinity resin (lane 7), His-Select washed with 50 column volumes (CV) 20mM imidazole buffer (lane 8), His-Select washed with 50 CV 20mM imidazole buffer, 20 CV 50mM imidazole buffer and 20 CV 80mM imidazole buffer (lane 9), Calmodulin Affinity resin (lane 10, same sample as lane 7), Anti-Protein C Affinity matrix (lane 11) and Strep-Trap™ (lane 12). Yields estimated for His_10_ and OneStrep based on Bradford staining was 8 mg / L HEK culture and 3 mg /L HEK culture respectively.

Protein purification from HEK293E cells in our hands is not very efficient using standard immobilized metal affinity purification. We therfore have introduced alternative C-terminal purification tags into pTT5 (Figure 3C). The *ccdB -* C - tag cassette into pTT5 increases plasmid size by 1350 bp. In order to analyze if this has an impact on transient gene expression in HEK293E cells, we compared expression levels of both intracellular eGFP and secreted CD40 ligand protein [18]. Both proteins were expressed from the original pTT vectors [2] and their respective pCoofy derivatives. Transient transfection of both genes show comparable levels of eGFP in the total cell lysate and CD40 ligand in the culture supernatant when either expressed from pTT or pCoofy (Figure 7A).

In order to test these alternative purification tags we fused them to eGFP, transiently expressed in HEK293E cells and purified with the respective affinity resin, except Æ54CPD54, which is specifically activated by inositol hexakisphosphate (InsP6) present in eukaryotic cells [19]. Comparison of C-terminal S-tag, His_10_, HPC, CBP and OneStrep showed best expression levels for both S and His_10_ tag. Protein yield was lowest for S-tag, HPC and CBP. Best yield and purity were obtained for eGFP-One Strep and eGFP-His_10_ when washed stringently with 50mM and 80mM imidazole (Figure 7B). In summary, we have shown effective protein expression and purification from pCoofy40 vectors that can now be included in our parallel cloning strategy.

## Conclusions

We have developed a method that allow one to tailor any given expression vector for efficient, fast, robust and cost-effective parallel cloning. High cloning efficiency is guaranteed via strong constitutive *ccdB* expression that, in contrast to DpnI digestion, is 100% efficient in counterselection of parental insert-less vector. The procedure is very robust and has been easily implemented in research groups in-house or externally. We have generated more than 30 parallel vectors for expression in bacteria, insect and HEK293 cells with different purification and solubility enhancing tags that we consider to be helpful in our workflow. This list of pCoofys has fundamentally increased our throughput and success rate in protein purification. We are constantly expanding the list of vectors and have also integrated the ccdB-C-tag cassette into Baculovirus, Pichia pastoris and Hansenula polymorpha vectors which still need to be validated.

With the strategy presented here it is straightforward to assemble any tag combination of interest for any selected application. Moreover, with the use of SLIC, as many as five inserts can be assembled in one reaction simultaneously with great efficiency [20]. Thus a modular combination of any vector element such as purification tag, signal sequences, antibiotic resistance etc. would be possible. The Llp-ccdB counterselection gene presented in this work could also enhance cloning efficiency of other cloning methods as RF or others.

## Methods

### Vector construction

Molecular biology methods were based on standard protocols. *E.coli* chemocompetent *ccdB* survival cells and OmniMAX™ 2 T1^R^ cells (Life Technologies, Darmstadt, Germany) were used for propagating *ccdB* plasmids and for transforming cloning reactions, respectively. PCR primers were ordered at Metabion (Martinsried, Germany). PCR was performed in 50 μl reaction mixes using high fidelity Phusion polymerase (NEB, Frankfurt, Germany). PCR products were analyzed on agarose gels and purified with High Pure PCR cleanup kit (Roche, Mannheim, Germany). Plasmid DNA was prepared using NucleoBond® or NucleoSpin® Plasmid Kits (Macherey Nagel, Düren, Germany). pETM vectors were provided by the EMBL [8], pTT by Yves Durocher [11] and pFastBac was purchased at Life Technologies. Synthetic *ccdB* DNA containing the promoter, Shine-Dalgarno and the coding sequence of *ccdB* was synthesized by Sloning BioTechnology (now part of Morphosys). The 2^nd^ generation cassette was synthesized by GeneArt® (Regensburg, Germany, now Life Technologies).

**pCoofy*E.coli*expression vectors** were generated by ligating the Llp5-*ccdB* HindIII restriction enzyme fragment from pPCRSript-ccdB into HindIII linearized pETM vectors. Additional tags, that were not present in the EMBL vector series, were added to the list: His6 was extended to His10 with the use of primer extensions; the OneStrep tag was PCR amplified from pPSG-IBA103-eGFP (IBA, Göttingen, Germany), the S tag was amplified from pET29 (Novagen, Darmstadt, Germany) and the Halo tag was amplified from pFN18a (Promega, Mannheim, Germany) template DNA**. The pCoofy transient insect expression vector** was derived from pIEX1. Vector was digested with XcmI and NotI and ligated to llp5-*ccdB*, that was PCR linearized with XcmI / NotI primer extensions. **The pCoofy Baculovirus expression vectors** were derived from pFastBac1. The sequence spanning N tag - 3C - Llp5-*ccdB* was PCR amplified using pCoofy1, pCoofy3 and pCoofy4 as template DNA to generate pCoofy27, pCoofy28, pCoofy29, respectively. PCR fragments were extended by RsrII /XhoI restriction sites and ligated into pFastBac1 linearized with RsrII /XhoI. **The pCoofy HEK expression vector** was derived from pTT5. The vector was linearized with EcoRI / NotI and ligated to the *ccdB -* C - tag cassette, which was PCR amplified with EcoRI / NotI primer extensions. Prior to use, the integrity of all vectors was verified by DNA sequencing. *ccdB* toxicity is a prerequisite for efficient counterselection and was verified for each new vector and for each individual vector preparation by transformation into *ccdB* non-resistant OmniMAX™ 2 T1^R^ cells. Functionality in cloning and expression was controlled for each new vector by eGFP SLIC cloning and small scale test expression in the appropriate host.

### SLIC cloning

All vectors were PCR linearized with their corresponding LP1 and LP2 primer combination, purified and stored at −20°C ready-to-use. Briefly, a 50 μl reaction mix containing 25 ng vector DNA, 50 pmol of each primer 0,4mM dNTP Mix, 1 Unit Phusion Polymerase and 1x Phusion Polymerase buffer was used in a 3-step PCR reaction: 1) 98°C for 3 min, 1 cycle; 2) 98°C for 30 sec followed by 72°C for 90 sec; 30 cycles; 3) 72°C for 10 min, 1 cycle. The PCR product is purified using High Pure PCR Cleanup Kit (Roche, Germany). The insert is PCR linearized with primers composed of gene specific sequences adjusted to a T_m_ of 56°C - 60°C plus 15 to 25 bp extensions complementary to LP1 and LP2 vector primer. PCR conditions need to be optimized for the respective gene. Typically 50 ng template DNA, 50 pmol of each primer 0,4mM dNTP Mix, 1 Unit Phusion Polymerase and 1x Phusion Polymerase buffer was used in a 3-step PCR reaction: : 1) 96°C for 5 min, 1 cycle; 2) 96°C for 30 sec followed by 50°C for 30 sec followed by 72°C for about 1min/kb; 30 cycles; 3) 72°C for 10 min, 1 cycle. The combination of LP1 and LP2 extensions has to be appropriate for the desired vector. The main vector series uses 3C-LP1 and ccdB-LP2. Alternative LP1 primers are used for N-terminal Sumo1, Sumo3 or no N tag at all. ccdB - LP2 is used in case no C-tag is introduced. It is important to include a stop codon in the insert LP2 primer, otherwise translation will proceed two codons (Arginine – Histidine) further downstream until the first stop codon in the LP2 primer extension is encountered. Diverse LP2 primers enable the introduction of certain C-terminal tags (Table 2). For expression in HEK cells a Kozak CCACC sequence should be added upstream the first ATG start codon in the LP1 gene specific primer sequence. A standard PCR program over 30 cycles including the final extension step was used. In the SLIC reaction, 100 ng of vector is mixed with insert at a molar ratio of 1:3. 1 μl recA protein (2 μg/ml stock) plus 1 μl recA buffer (10x) (NEB, Germany) was added to a final reaction volume of 10 μl, incubated for 30 min at 37°C and transformed into chemocompetent OmniMAX™ 2 T1^R^ cells with slightly extended incubation times: 30 min on ice, 1 min heat shock at 42°C, 5 Typically, three clones per construct were selected and sequenced prior to use in expression.

### Protein expression in *E.coli*

Expression plasmids were transformed into *E.coli* BL21 (DE3) Rosetta expression strains. Precultures were grown overnight in yeast extract supplemented with 3,5% glycerol (YG) at 30°C, 130 rpm. Expression cultures were either inoculated with colonies from agar plate or with preculture to a final oD_600_ of 0,004 and grown at 2 ml volume in 24 deepwell plates (Whatman, GE Healthcare, Germany) covered with airpore tape sheets (Qiagen, Hilden, Germany). Cultures were grown in autoinduction medium [21] at 24 or 30°C for 24h or at 37°C overnight. 18°C expression cultures in YG medium were first grown at 37°C, induced with 0,5 – 1mM IPTG and shifted to 18°C for over night expression. Cultures were pelleted, resuspended in 200 μl buffer A (50 mM Na-Phosphate pH 8,0, 500 mM NaCl, 10% glycerol, 10 mM imidazole) including protease inhibitors AEBSF-HCl (1 mM), Aprotinin (2 μg/ml), Leupeptin (1 μg/ml) Pepstatin (1 μg/ml) and lyzed mechanically in a cooled Tissue Lyzer (Retsch, Hann, Germany) with glass beads (Sigma, Munich, Germany) at 30 Hz for 5 min. Cell extracts were centrifuged for 30 min at 14.000 rpm at 4°C.

### Protein expression in insect cells

HighFive and SF9 suspension cultures were grown in ExCell405 and ExCell420 medium, respectively (Sigma, Munich, Germany) at 27°C in 50 mm Unitron shakers (Infors, Bottmingen, Switzerland). 2 ml test expression cultures were shaken in 25 ml polystyrene screw cap tubes (Sarstedt, Nümbrecht, Germany) at 120 rpm. Cell viability and cell size were monitored on a Vi-CELL® instrument (Beckman Coulter, Krefeld, Germany).

Baculovirus expression was performed according to the Bac-to-Bac® protocol (Life Technologies, Darmstadt, Germany). Bacmid transfected SF9 cells were typically harvested after 4–5 days, at maximum cell size and onset of cell lysis. The titer of this first virus stock was determined with the SF9 easy titer cell line [22]. Virus was either amplified in 2 subsequent steps to generate P1 and P2 virus stock or used to generate **B**aculo **I**nfected **I**nsect **C**ells (BIIC) as described previously [23]. For test expression, High Five and SF9 cells were infected at 1 × 10^6^ cells / ml with virus stock or BIIC at different dilutions, typically in the range of 1:1000 – 1:10.000 and harvested after different time points. Viability and cell size was recorded for every expression culture.

### Protein expression in HEK293E cells

HEK293E suspension cells were grown in serum free Freestyle medium (Life Technologies, Darmstadt, Germany) supplemented with G418 to 1 μg/ml and Pluronic to 0,1% at 37°C in 50 mm Unitron CO_2_ (5%) shakers (Infors, Bottmingen, Switzerland) Transient transfection was performed as described [24]. Briefly, 10 ml suspension cultures in 125 ml Erlenmeyer flasks were adjusted to a cell density of 1x10^6^ cells/ml. 1 μg plasmid DNA per ml culture was diluted in 100 μl PBS and mixed with 2 μl PEI (25 kDa linear, 1mg/ml, Polysciences). After 15 min incubation at room temperature the mix was added dropwise to the cells. For secreted proteins, A25 trypton (OrganoTechnie, La Courneuve, France) was added to a final concentration of 0,5% 24 h after transfection. Cells were harvested after 72, lyzed in respective binding buffers including protease inhibitors AEBSF-HCl (1 mM), Aprotinin (2 μg/ml), Leupeptin (1 μg/ml) Pepstatin (1 μg/ml) and lyzed mechanically in a Dounce homogenizer. Cell extracts were centrifuged for 30 min at 14.000 rpm at 4°C. Depending on the C-terminal tag fused to the protein, lysate supernatants were loaded on the following affinity resins: His-Select (Sigma, Munich, Germany); Calmodulin (Stratagene, Agilent Technologies, Germany), S-Protein (Novagen, Darmstadt, Germany), Anti-Protein C Affinity Matrix (Roche, Mannheim, Germany) and StrepTrap™ HP (GE Healthcare, Germany). Protein binding, washing and elution was performed according to the manufacturers instructions.

### Protein detection

All proteins samples were analyzed on 4–12% SDS-PAGE gradient gels (NuPAGE in MES buffer system, Life Technologies, Darmstadt, Germany), visualized by Coomassie staining, Silver staining or on P80 and P230 BioAnalyzer2100 Chips (Agilent, Böblingen, Germany). For western blot analysis, proteins were blotted on Immobilon-P PVDF (Millipore, Schwalbach, Germany) and visualized with HisProbe™-HRP (Thermo Scientific, Munich, Germany).

## Abbreviations

PCR: Polymerase chain reaction;SLIC: **S**equence and **L**igation **I**ndependent **C**loning;Bp: Basepairs;HRV: Human rhinovirus;GFP: Green fluorescent protein;HTTP: High throughput;RF cloning: Restriction free cloning;OmpA: Outer membrane lipoprotein A;SUMO: Small Ubiquitin-like Modifier;GOI: Gene of interest

## Competing interests

The authors declare that they have no financial and no non-financial competing interests.

## Authors’ contributions

JS constructed all pCoofys except pCoofy27,28 and 29, SLIC cloned inserts and performed insect cell expressions. HB constructed pCoofy27, 28, 29 and pETM28-Sumo1 and Sumo3. CS SLIC cloned inserts and performed bacterial expressions. SS designed the cloning strategy and vectors, performed bacterial, insect and mammalian expression experiments and wrote the manuscript. All authors read and approved the final manuscript.
